# Functional outcomes following injury in centenarians: a nationwide retrospective observational study

**DOI:** 10.1186/s13017-025-00595-6

**Published:** 2025-04-04

**Authors:** Ryo Yamamoto, Brian J. Eastridge, Ramon F. Cestero, Keitaro Yajima, Akira Endo, Kazuma Yamakawa, Junichi Sasaki

**Affiliations:** 1 Trauma Service/Department of Emergency and Critical Care Medicine, Keio University School of Medicine, 35 Shinanomachi, Shinjuku, Tokyo 160-8582 Japan; 2 Department of Surgery, UT Health San Antonio, 7703 Floyd Curl Drive, San Antonio, TX 78229-3900 USA; 3 Department of Acute Critical Care Medicine, Tsuchiura Kyodo General Hospital, 4-1-1 Otsuno, Tsuchiura, Ibaraki 300-0028 Japan; 4 Department of Emergency and Critical Care Medicine, Osaka Medical and Pharmaceutical University, 2-7 Daigakumachi, Takatsuki, Osaka 569-8686 Japan

**Keywords:** Older patients, Elderly, Physical function, Survivor

## Abstract

**Background:**

Advances in healthcare and the development of various technologies have improved disease-free longevity. Although the number of healthy centenarians is gradually increasing, studies on postinjury functions among centenarians are lacking. Therefore, we aimed to determine the clinical predictors of mortality and unfavorable functions after injury among centenarians.

**Method:**

A retrospective study was conducted using a nationwide trauma database, and data from patients aged ≥ 100 years across ≥ 250 institutions during 2019–2022 were analyzed. Patient demographics, comorbidities, mechanism of injury, injury severity, vital signs on arrival, and pre- and in-hospital treatments were compared between survivors and non-survivors as well as between survivors who had and did not have the ability to live independently at discharge, which was defined as Glasgow Outcome Scale (GCS) score of ≤ 3. Independent predictors of in-hospital mortality and unfavorable functions after injury were examined using a generalized estimating equation model to account for institutional and regional differences in the management and characteristics of centenarians.

**Results:**

Of the 409 centenarians, 384 (93.9%) survived to discharge. Although 208 (50.9%) patients had lived independently before the injury, only 91 (22.2%) could live independently at discharge. All patients had blunt injury, and fall from standing was the most frequent (86.6%) mechanism. The injury severity score was 10 ± 5, and surgery/angiography was performed in < 2% of the centenarians, except for fracture fixation in the extremity/pelvis, which was conducted in 225 (55.0%) patients. The adjusted model revealed three independent predictors of in-hospital mortality: male gender, mechanism of injury other than fall from standing, and GCS score on arrival. In contrast, only injury severity in the extremity/pelvis was an independent predictor of unfavorable functions after injury.

**Conclusion:**

Male gender, mechanisms of injury other than fall from standing, and GCS on arrival were associated with higher in-hospital mortality. Injury severity in the extremity/pelvis was related to dependent living after injury among centenarians.

**Supplementary Information:**

The online version contains supplementary material available at 10.1186/s13017-025-00595-6.

## Background

Advances in healthcare and the development of various technologies have improved the field of preventive medicine and disease-free longevity [1]. Although life expectancy varies across countries/regions depending on socioeconomic status [2], the number of healthy centenarians is gradually increasing globally [3]. Notably, early recovery from severe diseases and tailored rehabilitation can sustain physical functions even among centenarians with several comorbidities [4].

Although optimal physical abilities help maintain the quality of life of centenarians [5], independent living can be a risk factor for injury, which is an emerging medical concern in matured health systems [6]. A recent single-center observational study involving 13 centenarians admitted due to trauma within the past 3 years showed that 12 of them could walk with a frame or stick before injury [7]. Another retrospective study involving 69 centenarians with hip fractures showed that > 50% of them could walk independently without supervision before injury [8]. Although there is limited evidence suggesting an increase in trauma incidence due to walking [9], the occurrence of injury during normal daily activities may be common among centenarians.

However, studies on postinjury outcomes, specifically functional outcomes, among centenarians are lacking. Most previous studies have been conducted among a small number of patients, with mortality being the only targeted outcome [10–12]. A retrospective study involving 474 injured nonagenarians/centenarians (aged ≥ 90 years) extensively examined patient characteristics and cumulative mortalities. Although head and spine injuries, high injury severity, and mechanical ventilation use were identified as predictors of mortality, functional outcomes were not provided or analyzed [11]. Considering that centenarians have unique physiological features, such as vulnerability to chronic inflammation and infectious diseases [13, 14], it is necessary to determine the clinical consequences after injury, including functional abilities, in this specific population.

Using a nationwide trauma database, we examined patient, injury, and treatment characteristics associated with mortality and unfavorable functions after injury among centenarians. We aimed to determine the clinical predictors of mortality and unfavorable functions after injury among centenarians.

## Methods

### Study design and setting

We conducted a retrospective cohort study using the Japan Trauma Data Bank (JTDB), which was established as a nationwide trauma registry in 2003 and maintained by the Japanese Association for the Surgery of Trauma (JAST) and the Japanese Association for Acute Medicine [15]. The JTDB database includes data from > 250 tertiary care centers, with most high-level tertiary care centers participating in the JTDB. The current research involving humans was approved by the institutional review boards of all participating hospitals. The need for informed consent was waived because of data anonymity.

The management protocol for trauma patient resuscitation in Japan, including the older population, was developed by the JAST based on the Advanced Trauma Life Support training program of the American College of Surgeons. As some hospitals in Japan do not always have trauma surgeons, emergency physicians sometimes independently decide and conduct the initial resuscitation. Japan is one of the countries with the longest life expectancy [16]; thus, trauma among centenarians is not rare and it is seen in most tertiary care centers without consultation with a geriatric physician.

### Study population

We reviewed data from the JTDB between 2019 and 2022. We included patients (1) who were aged ≥ 100 years and (2) admitted due to nonburn injury. Moreover, we excluded those without measurable blood pressure or signs of life (pupillary response, spontaneous ventilation, extremity movement, or cardiac electrical activity) upon hospital arrival.

### Data collection and definition

JTDB data included age; gender; physical disability before injury; mechanism of injury; vital signs on arrival; Abbreviated Injury Scale (AIS) score; Injury Severity Score (ISS); prehospital treatments, including intubation, fluid administration, and transfusion; presence of a physician at the prehospital stage; transportation time; resuscitative procedures after hospital arrival, including surgery/angiography; length of intensive care unit stay; length of ventilator use; survival status at discharge; and Glasgow Outcome Scale (GOS) at discharge.

### Outcome measures

GOS was used to evaluate functional outcome at discharge, which was the primary outcome [17]. Unfavorable and favorable functional outcomes were defined as GOS scores of ≤ 3 and ≥ 4, respectively (1, dead; 2, vegetative state and unable to interact; 3, severe disability and unable to live independently; 4, moderate disability and unable to resume previous social activities; 5, good recovery and able to resume previous social activities).

### Statistical analysis

Patient, injury, treatment, and outcome characteristics were compared between survivors and non-survivors at discharge and between survivors who had and did not have unfavorable functional outcomes. Characteristics related to in-hospital mortality and unfavorable functional outcomes (GOS ≤ 3) were analyzed using multivariate logistic regression analyses fitted with a generalized estimating equation (GEE) model [18], accounting for within-institution clustering to consider institutional and regional differences in the management of centenarians with injury. In the GEE model, relevant potential covariates were selected from known predictors of clinical outcomes in patients with trauma and older individuals based on previous studies [11, 19–21]. To account for the limited sample size, we selected covariates in the final GEE models from the patient, injury, and treatment variables that showed a difference in univariate analyses.

Two sensitivity analyses were performed to validate the predictors of in-hospital mortality and unfavorable functional outcomes. First, as endogeneity between the age of ≥ 100 years and low tendency to provide invasive treatment is expected, GEE models without treatment variables after hospital arrival were developed, and predictors of in-hospital mortality and unfavorable function were examined. Second, considering the low number of included patients, logistic regression models with bootstrapping (the model is resampled 1,000 times) were developed [22] using the same variables in the primary analysis.

Descriptive statistics were presented as means (standard deviations), medians (interquartile ranges), or numbers (percentages). In hypothesis testing, significance was considered at a two-sided α-threshold of 0.05. In the variable selection for the final GEE models, an α threshold of 0.1 indicated a significant difference in univariate analyses. The association between each clinical factor and in-hospital mortality or unfavorable functional outcome was presented as odds ratio (OR) with a 95% confidence interval. All statistical analyses were performed using Statistical Package for the Social Sciences (version 29.0; IBM Corp., Armonk, NY, USA).

## Results

### Patient characteristics

**Fig. 1:**
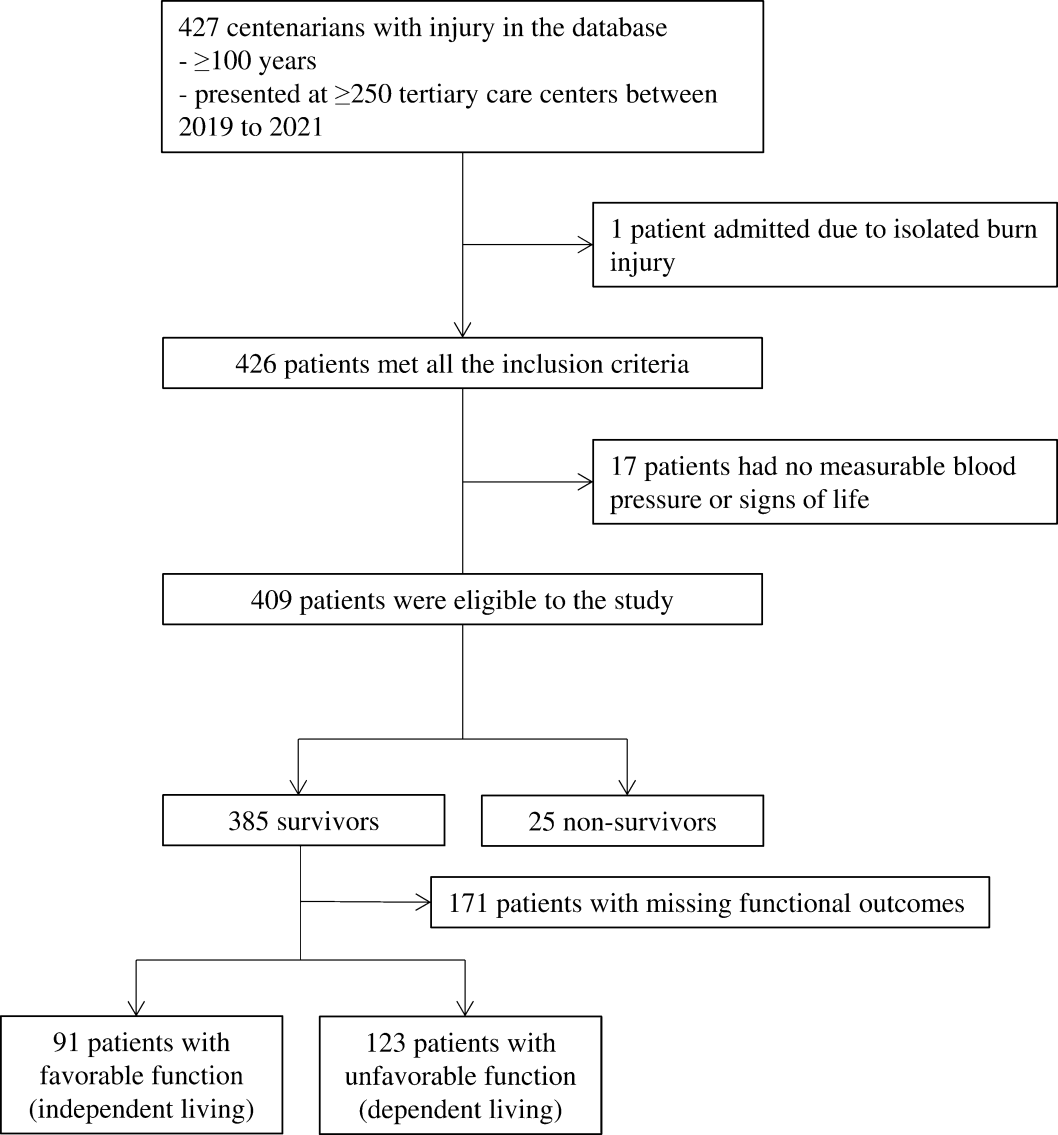
Patient flow diagram. Among 427 centenarians with injury in the database, 409 had measurable blood pressure and vital signs and were admitted due to nonburn injury, making them eligible for this study. A total of 385 centenarians (94.1%) survived at discharge, and 91/214 (42.5%) patients had favorable function with abilities to live independently at discharge

Among 427 centenarians with injury in the database, 409 had measurable blood pressure with vital signs and were admitted due to nonburn injury, making them eligible for the study (Fig. 1). Moreover, 385 (94.1%) centenarians survived at discharge, and 91/214 (42.5%) patients had favorable function with abilities to live independently at discharge.

Table 1 shows patient, injury, treatment, and outcome characteristics of survivors and non-survivors at discharge. Survivors had higher GCS and lower respiratory rate (RR) on arrival (14 [13–15] vs. 11 [5–14] and 19/min [16–21] vs. 22/min [17–26]), lower AIS in the head/neck, and higher AIS in the extremity/pelvis (0 [0–0] vs. 3 [0–5] and 3 [3] vs. 2 [0–3]) than non-survivors. Furthermore, a higher number of survivors experienced fall from standing and underwent surgery in the extremity/pelvis (339 [88.3%] vs. 16 [64.0%] and 222 [57.8%] vs. 3 [12.0%]).

**Table 1:** Characteristics of centenarians with trauma

	Survivor		Nonsurvivor		*p*-value
Case	384		25		
Age, years, median (IQR)	101	(100–102)	101	(100–102)	0.923
Gender, male, n (%)	48	(12.5%)	9	(36.0%)	**0.001**
Charlson comorbidity index, median (IQR)	1	(0–1)	1	(1–2)	0.116
Physical disability before injury, n (%)	192	(53.8%)	16	(72.7%)	0.083
Mechanism of injury, blunt, n (%)	374	(100.0%)	21	(100.0%)	–
Fall—from standing, n (%)	339	(88.3%)	16	(64.0%)	** < 0.001**
Fall—others, n (%)	23	(6.0%)	3	(12.0%)	0.206
Vital signs on hospital arrival, median (IQR)					
GCS	14	(13–15)	11	(5–14)	** < 0.001**
SBP, mmHg	151	(131–169)	147	(95–170)	0.289
HR, /min	80	(70–90)	85	(68–108)	0.325
RR, /min	19	(16–21)	22	(17–26)	**0.049**
SpO_2_, %	97	(95–98)	97	(95–99)	0.633
AIS, median (IQR)					
Head/neck	0	(0–0)	3	(0–5)	** < 0.001**
Face	0	(0–0)	0	(0–0)	0.526
Chest	0	(0–0)	0	(0–0)	0.523
Abdomen	0	(0–0)	0	(0–0)	0.895
Extremity/Pelvis	3	(3–3)	2	(0–3)	**0.003**
Body surface	0	(0–0)	0	(0–0)	0.256
ISS, median (IQR)	9	(9–9)	16	(9–25)	** < 0.001**
Prehospital procedure, n (%)					
Intubation	0	(0.0%)	0	(0.0%)	–
Fluid administration	5	(1.3%)	3	(12.0%)	**0.009**
Transfusion	0	(0.0%)	0	(0.0%)	–
Physician presence at prehospital	2	(0.5%)	0	(0.0%)	0.880
Transportation time, min, median (IQR)	27	(21–37)	25	(19–35)	0.460
Surgery/angiography, n (%)					
Head	6	(1.6%)	1	(4.0%)	0.359
Neck	0	(0.0%)	0	(0.0%)	–
Face	1	(0.3%)	0	(0.0%)	0.939
Chest	0	(0.0%)	0	(0.0%)	–
Abdomen	0	(0.0%)	0	(0.0%)	–
Extremity/pelvis	222	(57.8%)	3	(12.0%)	** < 0.001**
Body surface	1	(0.3%)	0	(0.0%)	0.939
Clinical outcomes, days, mean (SD)					
Length of ICU stay	1	(2)	1	(2)	0.892
Ventilator use	0	(3)	0	(0)	0.501

Bold values indicate the significance is defined as* p*-value < 0.05

IQR, interquartile range; GCS, Glasgow coma scale; SBP, systolic blood pressure; HR, heart rate; RR, respiratory rate; SpO_2_, peripheral oxygen saturation; AIS, Abbreviated Injury Scale; ISS, Injury Severity Score; ICU, intensive care unit

Table 2 shows the characteristics of centenarians who survived with and without favorable functional outcomes. Compared with those without favorable function, centenarians with favorable function had higher AIS in the head/neck, chest, and abdomen and lower AIS in the extremity/pelvis (3 [0–3] vs. 3 [3]). Moreover, centenarians with abilities to live independently at discharge underwent surgery in the extremity/pelvis less frequently (46 [50.5%] vs. 87 [70.7%]).

**Table 2:** Characteristics of survivors

	Independent living		Dependent living		*p*-value
Case	91		123		
Functional status at discharge, n (%)					
Able to resume previous social activities	50	(54.9%)	–		–
Unable to resume previous social activities	41	(45.1%)	–		–
Unable to live independently	–		122	(99.2%)	–
Unable to interact	–		1	(0.8%)	–
Age, years, median (IQR)	101	(100–102)	101	(100–102)	0.300
Gender, male, n (%)	11	(12.1%)	10	(8.1%)	0.336
Charlson Comorbidity Index, median (IQR)	1	(0–1)	1	(0–1)	0.299
Physical disability before injury, n (%)	50	(56.2%)	80	(65.6%)	0.107
Mechanism of injury, blunt, n (%)	88	(100.0%)	118	(100.0%)	–
Fall—from standing, n (%)	78	(85.7%)	109	(88.6%)	0.527
Fall—others, n (%)	8	(8.8%)	7	(12.0%)	0.380
Vital signs on hospital arrival, median (IQR)					
GCS	14	(13–15)	14	(13–15)	0.515
SBP, mmHg	155	(134–171)	153	(133–170)	0.450
HR, /min	80	(72–91)	80	(70–88)	0.711
RR, /min	20	(17–23)	18	(16–20)	0.051
SpO_2_, %	97	(94–98)	96	(94–98)	0.226
AIS, mean (SD), median (IQR)					
Head/Neck	1 (1),	0 (0–0)	0 (1),	0 (0–0)	**0.026**
Face	0 (0),	0 (0–0)	0 (0),	0 (0–0)	0.182
Chest	0 (1),	0 (0–0)	0 (0),	0 (0–0)	**0.001**
Abdomen	0 (1),	0 (0–0)	0 (0),	0 (0–0)	**0.041**
Extremity/Pelvis	2 (1),	3 (0–3)	3 (1),	3 (3–3)	** < 0.001**
Body surface	0 (0),	0 (0–0)	0 (0),	0 (0–0)	0.204
ISS, median (IQR)	9	(9–9)	9	(9–9)	0.577
Prehospital procedure, n (%)					
Intubation	0	(0.0%)	0	(0.0%)	–
Fluid administration	2	(2.2%)	1	(0.8%)	0.388
Transfusion	0	(0.0%)	0	(0.0%)	–
Physician presence at prehospital	1	(1.1%)	1	(0.8%)	0.668
Transportation time, min, median (IQR)	30	(22–37)	25	(19–39)	0.139
Surgery/angiography, n (%)					
Head	1	(1.1%)	2	(1.6%)	0.612
Neck	0	(0.0%)	0	(0.0%)	–
Face	0	(0.0%)	0	(0.0%)	–
Chest	0	(0.0%)	0	(0.0%)	–
Abdomen	0	(0.0%)	0	(0.0%)	–
Extremity/Pelvis	46	(50.5%)	87	(70.7%)	**0.003**
Body surface	1	(1.1%)	0	(0.0%)	0.425
Clinical outcomes, days, mean (SD)					
Length of ICU stay	0	(1)	1	(3)	0.070
Ventilator use	0	(0)	1	(5)	0.652

Bold values indicate the significance is defined as* p*-value < 0.05

IQR, interquartile range; GCS, Glasgow coma scale; SBP, systolic blood pressure; HR, heart rate; RR, respiratory rate; SpO_2_, peripheral oxygen saturation; AIS, Abbreviated Injury Scale; SD, standard deviation; ISS, Injury Severity Score; ICU, intensive care unit

### Clinical predictors of in-hospital mortality

To account for institutional differences in the management of centenarians, we developed the GEE model to predict in-hospital mortality, which included gender, physical disability before injury, mechanism of injury, GCS and RR on arrival, AIS in the head/neck and extremity/pelvis, ISS, prehospital fluid resuscitation, and surgery in the extremity/pelvis as covariates based on the results in univariate analyses (Table 3).

**Table 3:** Clinical factors associated with in-hospital mortality of centenarian

	p-value	OR (95% CI)	
Patient characteristics			
Male	**0.014**	**5.75**	**(1.43–23.26)**
Physical disability before injury	0.224	2.74	(0.57–10.64)
Severity of injury			
Fall from standing	** < 0.001**	**0.11**	**(0.03–0.37)**
GCS on hospital arrival, by one point decrease	**0.005**	**1.35**	**(1.09–1.66)**
RR on hospital arrival, by 1/min increase	0.167	1.04	(0.99–1.09)
AIS in head/neck, by one point increase	0.096	2.12	(0.88–5.11)
AIS in extremity/pelvis, by one point increase	0.211	1.65	(0.75–3.62)
ISS, by one point increase	0.287	0.91	(0.77–1.08)
Treatment			
Prehospital fluid administration	0.154	6.21	(0.51–76.92)
Surgery/angiography in the extremity/pelvis	0.057	0.05	(0.00–1.10)

Bold values indicate the significance is defined as* p*-value < 0.05

OR, odds ratio; CI, confidence interval; GCS, Glasgow coma scale; RR, respiratory rate; AIS, Abbreviated Injury Scale; and ISS, Injury Severity Score

This adjusted model revealed several clinical factors related to in-hospital mortality: male gender (OR for in-hospital mortality, 5.75 [1.43–23.26]; *p* = 0.014) and lower GCS on arrival (OR for in-hospital mortality, 1.35 [1.09–1.66]; *p* = 0.005). Conversely, fall from standing was associated with lower in-hospital mortality among centenarians (OR, 0.11 [0.03–0.37]; *p* < 0.001).

In the sensitivity analyses with another GEE model without treatment variables after hospital arrival and a logistic regression model with bootstrapping, male gender, GCS on hospital arrival, and mechanism of injury other than fall from standing were independent predictors of in-hospital mortality (Table S1).

### Clinical predictors of unfavorable functional outcomes

The final GEE model to predict dependency in daily life at discharge included physical disability before discharge; RR on hospital arrival; AIS in the head/neck, chest, abdomen, and extremity/pelvis; and surgery in the extremity/pelvis (Table 4). However, only AIS in the extremity/pelvis was associated with unfavorable functional outcomes following injury among centenarians (OR 1.98 [1.19–3.28]; *p* = 0.009). Moreover, sensitivity analyses showed that AIS in the extremity/pelvis was the only predictor of dependent living after injury (Table S1).

**Table 4:** Clinical factors associated with unfavorable function of centenarians

	*p*-value	OR (95% CI)	
Patient characteristics			
Physical disability before injury	0.693	1.18	(0.52–2.65)
Severity of injury			
RR on hospital arrival, by 1/min increase	0.546	0.98	(0.91–1.05)
AIS in head/neck, by one point increase	0.181	1.35	(0.87–2.09)
AIS in chest, by one point increase	0.353	0.79	(0.47–1.31)
AIS in abdomen, by one point increase	0.812	1.14	(0.39–3.36)
AIS in extremity/pelvis, by one point increase	**0.009**	**1.98**	**(1.19–3.28)**
Treatment			
Surgery/angiography in the extremity/pelvis	0.773	1.13	(0.50–2.56)

Bold values indicate the significance is defined as* p*-value < 0.05

OR, odds ratio; CI, confidence interval; RR, respiratory rate; and AIS, Abbreviated Injury Scale

## Discussion

This study identified that > 50% of centenarians had inadequate abilities to live independently after injury treatment, whereas > 90% could survive to discharge. In addition, injury severity in the extremity/pelvis was the only predictor of unfavorable function, whereas male gender, lower GCS, and mechanism of injury other than fall from standing were associated with increased in-hospital mortality.

Several beneficial features of the identified predictors should be considered for clinical implications. First, injury in the musculoskeletal system may highly disrupt the ability to live independently among centenarians. Although previous studies have reported that fracture fixation in the extremity/pelvis was associated with unfavorable outcomes [23, 24], our study indicated that this relationship might have been due to the confounding effects of injury severity. Thus, surgery should be performed as needed. Second, the physiological rather than anatomical severity of traumatic brain injury would be a significant predictor of in-hospital mortality among centenarians. Although AIS in the head was not independently associated with mortality in this study, GCS on arrival substantially affected survival to discharge, which was consistent with previous reports of a linear correlation between the initial GCS and mortality from TBI [25, 26]. Third, the mechanism of injury would be associated with in-hospital mortality but not with functional outcomes in centenarians. Although fall from standing was related to lower in-hospital mortality, a considerable number of centenarians did not regain adequate ability to live independently after fall from standing, indicating that this is a serious mechanism in this population.

Notably, among the centenarians who could live independently at discharge, > 50% had lived with physical disability before injury, which might be due to appropriate rehabilitation and optimal installation of assistive equipment at home during hospital stay [27, 28]. Therefore, we do not recommend limiting treatment based on preinjury physical disability. Conversely, one-third of the centenarians with dependency in daily life at hospital discharge did not have a physical disability before trauma. As this loss of capability would significantly impede the quality of life of healthy centenarians, further investigation should be conducted to manage the subpopulation of centenarians whose ideal physical function is impaired by severe extremity/pelvis injury.

Although several prognostic factors were identified for in-hospital mortality and functional consequences following trauma, we only examined survival status and functions at discharge. Considering that long-term outcomes, such as abilities for daily living a year after injury, are more clinically feasible as a goal of treatment, other predictors of favorable function may exist. However, because the age of ≥ 100 years is longer than the life expectancy in any countries/regions and treatment tends to be easily deviated from the standard [29], the identified predictors in this study might help in the decision-making process while treating centenarians with injury.

### Limitations

Our study results must be interpreted within the context of the study design. We analyzed data obtained from the JTDB, which does not record treatment details. Therefore, our results may differ with existing unmeasured confounding factors, such as patient preference and resources related to geriatric medicine. However, sensitivity analyses after excluding in-hospital treatment variables had similar results, wherein possible endogeneity between the age of ≥ 100 years and low tendency to provide invasive treatment in centenarians was minimized. Another limitation is the lack of data on strategies for rehabilitation. In addition to injury treatment, we could not evaluate the timing and intensity of rehabilitation, which were related to unfavorable functional outcomes. Furthermore, the database did not include ability for each daily activity, such as eating, dressing, toileting, and working. Although some of the abilities are more important depending on the living environment, we could not evaluate predictors of each ability. Moreover, the outcome used in this study was GOS, which is generally evaluated ≥ 6 months after injury. Considering that physical function gradually improves in some patients, unfavorable functional outcomes might have been overestimated. Although we used a nationwide database, the sample size was still limited due to the nature of the targeted population. The identified predictors of in-hospital mortality and unfavorable functional outcomes following injury should be validated using a large cohort of centenarians.

## Conclusions

Our study showed that > 50% of centenarians could not live independently at hospital discharge after injury treatment, whereas > 90% could survive. Injury severity in the extremity/pelvis was the only predictor of unfavorable functions after injury, whereas male gender, mechanisms of injury other than fall from standing, and GCS on arrival were associated with higher in-hospital mortality.

## Supplementary Information

Additional file 1.

## Data Availability

The study data are available from the Japanese Association for the Surgery of Trauma (JAST) and Japanese Association for Acute Medicine (JAAM); however, restrictions apply to the data availability, which are used under license for this study and are therefore not publicly available. However, data are available from the authors upon reasonable request with permission from the JAST and JAAM.

## List of abbreviations

AIS: Abbreviated Injury Scale
GCS: Glasgow Coma Scale
GEE: Generalized estimating equation
GOS: Glasgow Outcome Scale
ISS: Injury Severity Score
JAST: Japanese Association for the Surgery of Trauma
JTDB: Japan Trauma Data Bank
OR: Odds ratio
RR: Respiratory rate
