# Supplementary material for: Functional outcomes following injury in centenarians: a nationwide retrospective observational study
Source: World J Emerg Surg. 2025 Apr 4;20:28. doi: 10.1186/s13017-025-00595-6 (PMC11969837; doi:10.1186/s13017-025-00595-6)
Supplement: Supplementary file 1 — Additional file 1. [file 13017_2025_595_MOESM1_ESM.docx]

| Table S1. Functional predictors in sensitivity analysis | | | | | |
| --- | --- | --- | --- | --- | --- |
|  |  | Excluding in-hospital treatment, OR (95% CI) | | Logistic regression with bootstrapping*, OR (95% CI) | |
| For in-hospital mortality | |  |  |  |  |
|  | Male | 4.03 | (1.02–16.13) | 5.76 | (1.24–26.73) |
|  | Fall from standing | 0.10 | (0.02–0.42) | 0.11 | (0.03–0.46) |
|  | GCS on hospital arrival, by one point decrease | 1.20 | (1.01–1.43) | 1.35 | (1.07–1.69) |
| For unfavorable function (GOS ≤3) | |  |  |  |  |
|  | AIS in extremity/pelvis, by one point increase | 2.02 | (1.23–3.26) | 1.98 | (1.17–3.35) |
| OR, odds ratio; CI, confidence interval; GCS, Glasgow coma scale; GOS, Glasgow outcome scale; and AIS, Abbreviated Injury Scale. *Same covariates were entered as the primary generalizing estimating equation model. | | | | | |
|  |  |  |  |  |  |
